# EEG-Based Brain Network Analysis of Chronic Stroke Patients After BCI Rehabilitation Training

**DOI:** 10.3389/fnhum.2022.909610

**Published:** 2022-06-27

**Authors:** Gege Zhan, Shugeng Chen, Yanyun Ji, Ying Xu, Zuoting Song, Junkongshuai Wang, Lan Niu, Jianxiong Bin, Xiaoyang Kang, Jie Jia

**Affiliations:** ^1^Laboratory for Neural Interface and Brain Computer Interface, State Key Laboratory of Medical Neurobiology, Engineering Research Center of AI and Robotics, Ministry of Education, Shanghai Engineering Research Center of AI and Robotics, MOE Frontiers Center for Brain Science, Institute of AI and Robotics, Academy for Engineering and Technology, Fudan University, Shanghai, China; ^2^Department of Rehabilitation Medicine, National Clinical Research Center for Aging and Medicine, Huashan Hospital, Fudan University, Shanghai, China; ^3^Shanghai Jinshan Zhongren Geriatric Nursing Hospital, Shanghai, China; ^4^Ji Hua Laboratory, Foshan, China; ^5^Yiwu Research Institute of Fudan University, Yiwu, China; ^6^Research Center for Intelligent Sensing, Zhejiang Lab, Hangzhou, China; ^7^National Center for Neurological Disorders, Shanghai, China

**Keywords:** EEG, functional connectivity, BCI therapy, chronic stroke, motor function rehabilitation, brain network

## Abstract

Traditional rehabilitation strategies become difficult in the chronic phase stage of stroke prognosis. Brain–computer interface (BCI) combined with external devices may improve motor function in chronic stroke patients, but it lacks comprehensive assessments of neurological changes regarding functional rehabilitation. This study aimed to comprehensively and quantitatively investigate the changes in brain activity induced by BCI–FES training in patients with chronic stroke. We analyzed the EEG of two groups of patients with chronic stroke, one group received functional electrical stimulation (FES) rehabilitation training (FES group) and the other group received BCI combined with FES training (BCI–FES group). We constructed functional networks in both groups of patients based on direct directed transfer function (dDTF) and assessed the changes in brain activity using graph theory analysis. The results of this study can be summarized as follows: (i) after rehabilitation training, the Fugl–Meyer assessment scale (FMA) score was significantly improved in the BCI–FES group (*p* < 0.05), and there was no significant difference in the FES group. (ii) Both the global and local graph theory measures of the brain network of patients with chronic stroke in the BCI–FES group were improved after rehabilitation training. (iii) The node strength in the contralesional hemisphere and central region of patients in the BCI–FES group was significantly higher than that in the FES group after the intervention (*p* < 0.05), and a significant increase in the node strength of C4 in the contralesional sensorimotor cortex region could be observed in the BCI–FES group (*p* < 0.05). These results suggest that BCI–FES rehabilitation training can induce clinically significant improvements in motor function of patients with chronic stroke. It can improve the functional integration and functional separation of brain networks and boost compensatory activity in the contralesional hemisphere to a certain extent. The findings of our study may provide new insights into understanding the plastic changes of brain activity in patients with chronic stroke induced by BCI–FES rehabilitation training.

## Introduction

Stroke is a cerebrovascular disease with high morbidity, disability, and mortality (Sheorajpanday et al., 2011; Larivière et al., 2018). Patients are likely to suffer various degrees of functional impairment after the onset of stroke, among which motor dysfunction is one of the most significant disabling manifestations after stroke (Krueger et al., 2015). Motor dysfunction seriously affects the quality of life of patients with stroke and their families, and therefore stroke rehabilitation is essential. Currently, resources for stroke rehabilitation are focused on the prognosis of patients with stroke in the acute and subacute phases (Teasell et al., 2012). For patients with stroke in the chronic phase, which is more than 6 months after stroke (Bernhardt et al., 2017), a standardized outpatient regimen of exercise fails to effectively promote the recovery of motor function. One possible contributing factor is the neuromuscular adaptation to a standardized outpatient regimen of exercise in patients with chronic stroke (Teasell et al., 2014). When neuromuscular adaptation occurs, finding a treatment regimen that differs from that during rehabilitation can be beneficial in overcoming the adaptive state (Page et al., 2004). Several recent studies have shown that alternative or new treatment options, such as brain–computer interface (BCI) combined with external devices or other neuromodulation paradigms, can be effective in chronic stroke rehabilitation (Broetz et al., 2010; Ramos-Murguialday et al., 2013; Mukaino et al., 2014; Naros and Gharabaghi, 2017; Mohanty et al., 2018; Miao et al., 2020).

Brain–computer interface can directly measure brain activity and convert it into control signals of computers or external devices. The BCI used to overcome stroke-related motor paralysis can be broadly divided into two categories: assistive BCI and rehabilitative BCI (Soekadar et al., 2015). The assistive BCI is designed to continuously or permanently control the robotic device to assist in daily life activities. The rehabilitative BCI is meant to establish connections between the brain and the periphery (Pichiorri and Mattia, 2020) and induce neuroplasticity to facilitate motor recovery (Soekadar et al., 2011). BCI focuses on brain activity and can recognize and enhance motor-related brain activity (Hallett, 2007). Due to this ability to modify brain activity, BCI is considered a form of endogenous neuromodulation that can induce plastic remodeling of brain activity (Pichiorri and Mattia, 2020). By altering the brain activity, BCI can induce recovery of function. There are two common strategies for the application of the BCI technique in motor function rehabilitation of patients with chronic stroke. The first strategy is to drive external devices, such as robotic devices or functional electrical stimulation (FES), to assist in the execution of limb movements. This strategy can close the sensorimotor loop disrupted by the stroke event and re-establish connections between the central nervous system and the periphery (Pichiorri and Mattia, 2020). Several studies have shown that patients with chronic stroke who receive BCI-assisted robotic therapy can achieve greater motor gains compared to robotic therapy alone (Ramos-Murguialday et al., 2013; Keng et al., 2014; Frolov et al., 2017). Representative among these studies is a randomized controlled study conducted by Ramos-Murguialday et al. (2013) in 32 patients with chronic stroke. Their results showed that BCI-driven arm orthosis improved upper limb motor function more significantly in patients with chronic stroke than in a control group where movements of the arm orthoses occurred randomly. Similar results have been reported in studies of the BCI combined with the Haptic Knob (HK) robot (Keng et al., 2014) and the BCI-controlled exoskeleton (Frolov et al., 2017) for the rehabilitation of patients with chronic stroke. In addition, some studies have compared the differences in motor function of patients before and after intervention, and the results have shown that BCI-driven robotic devices play a beneficial role in the rehabilitation of patients with chronic stroke (Shindo et al., 2011; Takashi et al., 2014; Sun et al., 2017; Lu et al., 2020). Similar effective effects were also found in studies on the rehabilitation of patients with chronic stroke based on BCI-driven FES (Tabernig et al., 2018). A recent study by Biasiucci et al. (2018) showed that BCI-driven FES was more effective in inducing significant and durable motor recovery in patients with chronic stroke than sham FES. They pointed out that BCI combined with FES can promote significant functional recovery and purposeful plasticity.

Another strategy underlying the design of BCI for motor rehabilitation after chronic stroke is called the “brain-to-brain” strategy (Pichiorri and Mattia, 2020). The goal of this strategy is to combine BCI with other neuromodulation paradigms to boost neuroplasticity at the central nervous system level and improve motor function. Mrachacz-Kersting et al. (2016) combined BCI and non-invasive transcranial magnetic stimulation (TMS) in the rehabilitation of patients with chronic stroke. They found that patients showed clinically relevant and significant improvements in motor function after the intervention. According to this result, they pointed out the possibility of BCI-based neurofeedback system for efficient and targeted induction of plastic changes in the motor cortex. In addition, some studies combined transcranial direct current stimulation (tDCS; Ang et al., 2015) and transcranial alternating current stimulation (tACS; Naros and Gharabaghi, 2017) with BCI for the rehabilitation training of patients with chronic stroke, and the results showed that the enhancement of sensorimotor rhythm (SMR) was significantly improved.

The effectiveness of BCI-based interventions in chronic stroke rehabilitation has been demonstrated in the aforementioned studies. The clinical assessment scale is usually used to assess the functional recovery of patients with chronic stroke. However, the clinical scale only reflects external motor performance, and the neurological changes related to functional recovery in patients with chronic stroke induced by BCI rehabilitation intervention need to be further studied. Brain regions and their interactions can be modeled as brain networks that describe the efficient transmission of information in the brain (Jin et al., 2017). Therefore, brain network analysis plays an important role in analyzing and revealing the complex neural mechanism of the brain. Brain network analysis can analyze brain signals from a new perspective and help to understand the interaction between brain regions. In recent years, EEG-based brain network analysis has been used to explore the neurological changes related to functional recovery in patients with chronic stroke. Christian et al. (2006) constructed brain networks by computing EEG coherence to assess differences in cortical connectivity between well-recovered patients with chronic stroke and healthy subjects. The results showed that after stroke, connectivity in the stroke hemisphere decreased and connectivity in the contralesional hemisphere was relatively increased. Borich et al. (2016) constructed brain networks for patients with chronic stroke by computing the imaginary part of coherence (IPC) of EEG to assess changes in cortical connectivity induced by transcranial magnetic stimulation (TMS). They characterized changes in cortical connectivity through changes in connection weights between electrode pairs. Their study is the first to report the association between recovery (or lack) and abnormal interhemispheric interactions in patients with chronic stroke using TMS-EEG. Sun et al. (2021) built brain networks by computational EEG coherence to investigate intervention-specific markers of motor improvement in patients with chronic stroke. Their analysis is based on connection weights between electrode pairs. The results showed that interhemispheric connectivity in the delta, theta, and alpha bands, and contralesional connectivity in the beta band were associated with motor improvement. Biasiucci et al. (2018) constructed EEG-based brain networks by computing the short-term direct directional transfer function (SdDTF) to explore changes in brain connectivity before and after BCI intervention. Their assessment of connectivity changes is based on changes in connection weights between electrode pairs within the region of interest. The results showed increased functional connectivity between motor areas of the affected hemisphere in patients with chronic stroke after BCI intervention.

Based on the above literature survey, we found that in the brain network analysis of patients with chronic stroke, few studies combined global and local graph theory measures to comprehensively evaluate EEG-based brain network changes. The above studies are based on changes in the connection weights between electrode pairs to reflect changes in functional connectivity and fail to measure the properties of the brain network at both the global and local levels. A comprehensive analysis of neurological changes in the brain can help to understand the plastic changes in brain activity after stroke and during rehabilitation. The main objective of this study was to comprehensively and quantitatively investigate the neurological changes induced by BCI–FES rehabilitation training in patients with chronic stroke from the level of functional integration and separation using global and local graph theory measures. We hypothesized that BCI–FES rehabilitation training would improve motor function in patients with chronic stroke and cause positive changes in brain networks.

## Materials and Methods

### Subjects

Patients were recruited from Huashan Hospital affiliated to Fudan University and Shanghai Jinshan Zhongren Geriatric Nursing Hospital. Inclusion criteria were as follows: (1) ischemic or hemorrhagic stroke diagnosed by computer tomography or magnetic resonance imaging; (2) age between 60 and 90 years; (3) stroke onset was more than 1 year; (4) being able to sit in a chair for at least 1 h. Exclusion criteria were as follows: (1) patients with vision problems; (2) unilateral neglect; (3) allergic to conductive paste; (4) cannot complete basic treatment. Twenty-four elderly patients with chronic stroke were enrolled in the study and randomly allocated to the BCI–FES group (*n* = 12) and the FES group (*n* = 12). Baseline demographic data and clinical characteristics of patients are presented in Table 1. This study was approved by the Ethics Committee of Huashan Hospital (KY2014-266) and performed according to the Declaration of Helsinki. All the patients signed the informed consent.

**Table 1:** Baseline demographic data and clinical characteristics of patients.

**Patient**	**Age (year)**	**Diagnosis**	**Lesion site**	**Lesion side**	**Time since stroke (month)**	**FMA**	
						**Pre**	**Post**
BCI01	75–80	Ischemic	Cortical and subcortical	R	34	3	5
BCI02	75–80	Ischemic	Cortical	R	19	1	5
BCI03	80–85	Ischemic	Subcortical	L	74	25	28
BCI04	65–70	Ischemic	Cortical	R	12	12	12
BCI05	60–65	Ischemic	Subcortical	R	39	5	7
BCI06	65–70	Hemorrhagic	Cortical	R	12	4	6
BCI07	80–85	Ischemic	Cortical	R	145	32	35
BCI08	80–85	Ischemic	Cortical and subcortical	R	26	33	33
BCI09	80–85	Ischemic	Cortical and subcortical	L	14	52	53
BCI10	75–80	Hemorrhagic	Cortical and subcortical	R	16	30	30
BCI11	65–70	Ischemic	Cortical and subcortical	R	29	30	30
BCI12	70–75	Ischemic	Cortical and subcortical	R	40	3	5
FES01	80–85	Ischemic	Cortical	L	27	0	0
FES02	85–90	Ischemic	Subcortical	R	12	58	58
FES03	80–85	Ischemic	Subcortical	R	28	45	45
FES04	75–80	Ischemic	Subcortical	R	22	27	25
FES05	65–70	Ischemic	Cortical and subcortical	R	41	20	20
FES06	85–90	Ischemic	Subcortical	L	36	44	43
FES07	80–85	Ischemic	Cortical	L	44	2	2
FES08	75–80	Hemorrhagic	Subcortical	L	110	6	6
FES09	75–80	Ischemic	Cortical and subcortical	R	35	56	59
FES10	75–80	Ischemic	Cortical	L	180	31	30
FES11	80–85	Ischemic	Cortical and subcortical	L	30	24	24
FES12	70–75	Ischemic	Cortical and subcortical	L	32	30	30

*M, male; F, female; L, left; R, right*.

### Rehabilitation Training

The patients in the two groups were routinely treated with basic rehabilitation therapy, including exercise therapy and occupational therapy for 1 h/time, 5 times a week, for a total of 4 weeks. Patients in the BCI–FES group received BCI training for 40 min/time, 3 times a week for 4 weeks. Patients in the FES group received FES training for 30 min/time, 3 times a week for 4 weeks. The FES electrodes were placed in the extensor carpi radialis and extensor carpi ulnar muscles of the patient's upper limp and the stimulation intensity of FES was based on the feeling of the patient and the induction of wrist dorsiflexion movement. In the BCI–FES group, FES is triggered by BCI system induced by the motor imagery of the patients. The upper limb of Fugl-Meyer assessment and resting-state EEG data were collected before and after 1-month intervention.

**Figure 1:**
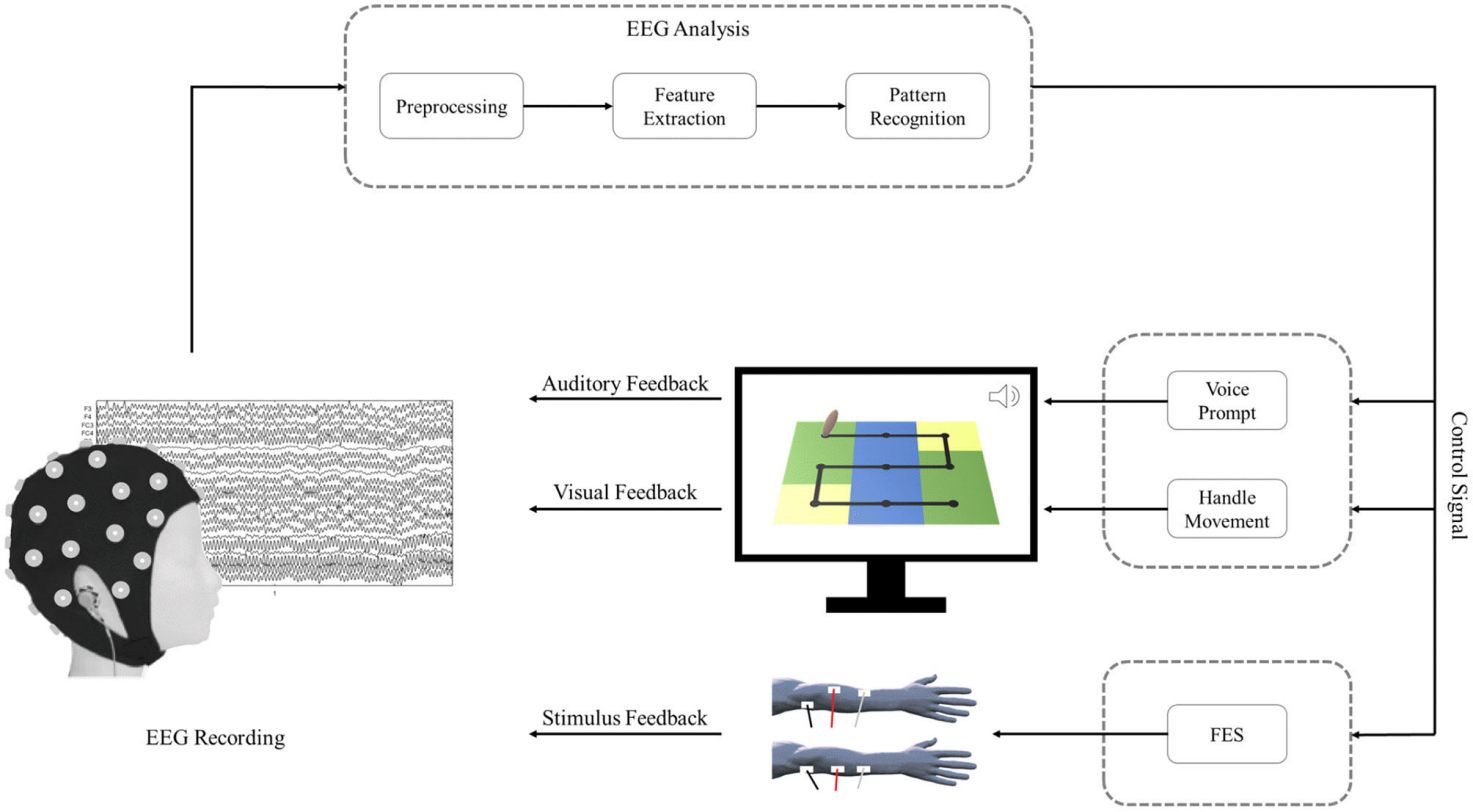
The schematic of the BCI–FES system.

The schematic of the BCI–FES system is shown in Figure 1. During BCI rehabilitation training, the patient's successful motor imagery of the affected hand is converted into control signals that drive feedback devices, including visual feedback, auditory feedback, and stimulus feedback. After a successful motor imagery task, the patient can hear voice prompts and observe handle movements on the screen. In addition, the control signal also drives the stimulator to deliver functional electrical stimulation to the patient.

**Figure 2:**
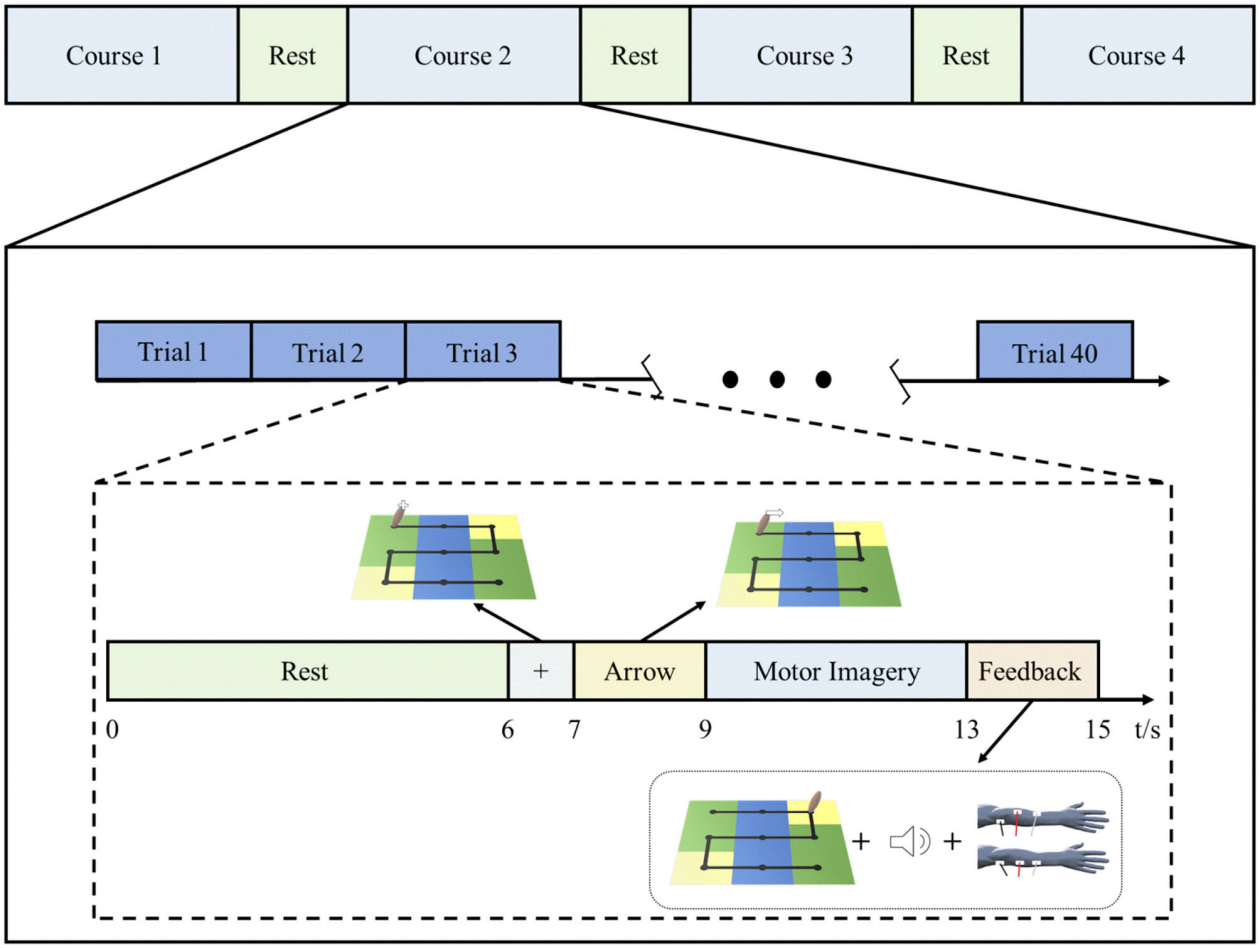
Rehabilitation training protocol of the BCI–FES system.

Each rehabilitation session for patients in the BCI–FES group included 4 courses, and each course contained 40 trials. The rest period after each course was determined according to the patient's tolerance and was generally 2–5 min. Figure 2 shows the rehabilitation training protocol of the BCI–FES system. At the beginning of each trial, the patient had a rest period of 6 s. At the end of the rest period, a white fixation cross was displayed on the handle for 1 s, indicating that the task is about to start. Then a white arrow appears on the handle to indicate the direction of movement of the motor imagery task. The arrow was present for 2 s. After the arrow disappeared, the patient imagined using the affected hand to move the handle. The motor imagery task lasted for 4 s, during which the patient kept the body still and avoided moving. At the end of the motor imagery, the patient received feedback. For successful motor imagery trials, patients heard a beeping sound (auditory feedback) and received a single electrical stimulus (stimulus feedback). For unsuccessful motor imagery trials, neither the beeping sound nor the electrical stimulation is present. The visual feedback is that the patient can observe the handle moving from the center of the current square, and the number of squares the handle moves depends on the motor imagery score.

### EEG Recordings and Preprocessing

Twenty-four electrodes placed in accordance with the international 10–20 standard system were used for EEG recordings. Before and after the intervention, we recorded EEG signals at a sampling frequency of 512 Hz for 10 min. For the first 5 min of EEG recording, the subject remained in an eye-closed resting state; after 5 min, the subject was asked to perform three elevated leg movements on the affected side, and for the last 5 min of EEG recording, the subject returned to an eye-closed resting state. In this study, only the first 5 min of EEG recordings were used for connectivity analyses.

Due to EEG artifact contamination or data loss, we finally used data from 17 of 24 patients with chronic stroke (BCI–FES = 8, FES = 9). Since the recording electrodes included two reference electrodes (A1 and A2), the reference electrodes were first removed during EEG pre-processing to obtain 22 channels (F3, F4, FC3, FC4, C3, C4, CP3, CP4, P3, P4, FT7, FT8, T3, T4, TP7, TP8, Fz, Oz, FCz, Cz, CPz, and Pz) of EEG data. Then all EEG recordings were filtered to the alpha band (8–13 Hz) by an FIR filter pass filter. After dividing the frequency band, we re-referenced the signals by average reference. The independent component analysis (ICA) was applied for the removal of EOG artifacts. All EEG preprocessing was performed based on EEGLAB toolbox of MATLAB.

Since the subjects had different injury hemispheres, for the consistency of analysis, the EEG data from patients with right hemisphere lesion were flipped. In this way, we uniformly defined the left hemisphere as the affected side and the right hemisphere as the unaffected side.

**Figure 3:**
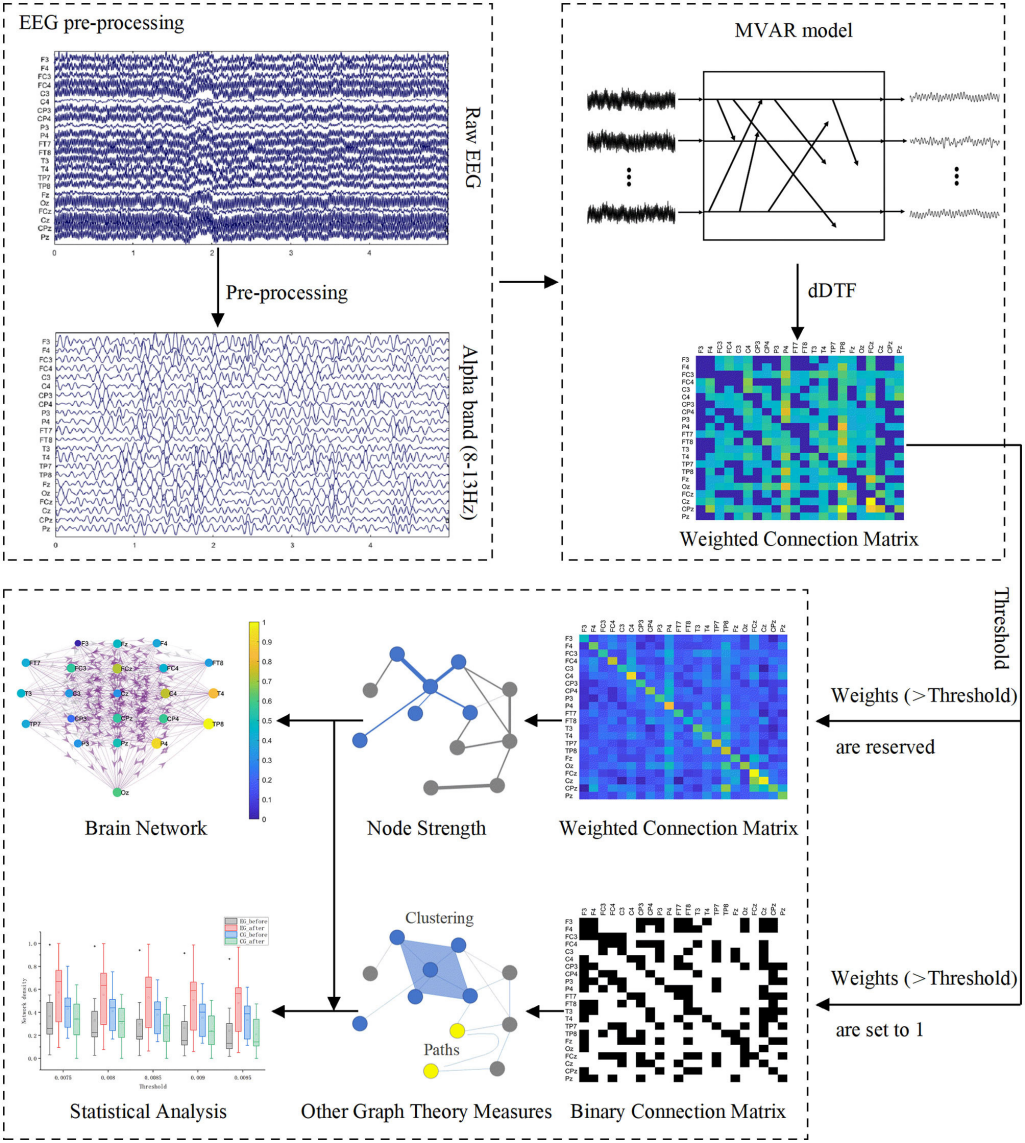
Schematic diagram of building functional brain networks based on EEG data using graph theory.

After preprocessing, we constructed functional brain networks based on EEG signals. To assess changes in the brain networks induced by BCI rehabilitation training, we also characterized brain networks using global and local graph theory measures. Figure 3 shows the schematic diagram of building functional brain networks based on EEG data using graph theory.

### EEG Functional Connectivity Measurement

Directed transfer function (DTF) is a multivariate effective connection measurement method based on Granger causality (Baccalá and Sameshima, 2001), which is often used to construct brain networks (De Vico Fallani et al., 2007). It is calculated on a multivariate autoregressive model (MVAR; Liu et al., 2010). For EEG data with k channels, its p-order MVAR model can be expressed as:


(1) $$\begin{array}{l} X\left(t\right)=\overset{p}{\underset{i=1}{\sum }}A\left(i\right)X\left(t-i\right)+E\left(t\right) \end{array}$$

where ***X***(***t***) represents the vector of the EEG signal at time t and ***p*** is the order of MVAR model. ***A***(***i***) are the model coefficients and ***E***(***t***) is the vector of white noise values. The model equations are transformed to the frequency domain:


(2) $$\begin{array}{l} X\left(t\right)=\;A^{-1}\left(f\right)E\left(f\right)=H\left(f\right)E\left(f\right) \end{array}$$

where ***H***(***f***) represents the transfer matrix of the system.

From the transfer matrix of the system, the normalized DTF is defined as:


(3) $$\begin{array}{l} \gamma_{ij}^{2}\left(f\right)=\frac{|H_{ij}\left(f\right){|}^{2}}{\sum_{n=1}^{k}|H_{in}\left(f\right){|}^{2}} \end{array}$$

where $\gamma_{ij}^{2}\left(f\right)$ denotes the proportion of the total information flowing into i from j to the total amount of information flowing into i.

The disadvantage of the DTF method is that in some cases it is not easy to distinguish between direct and indirect connections. To overcome this drawback, Korzeniewska et al. proposed an improved DTF method called direct directed transfer function (dDTF), which combines the advantages of DTF and partial coherences (Korzeniewska et al., 2003). The mathematical formulation for dDTF is as follows:


(4) $$\begin{array}{l} \delta_{ij}^{2}\left(f\right)=\eta_{ij}^{2}\left(f\right)P_{ij}^{2}\left(f\right) \end{array}$$

(5) $$\begin{array}{l} \eta_{ij}^{2}\left(f\right)=\frac{|H_{ij}\left(f\right){|}^{2}}{\sum_{f}\sum_{n=1}^{k}|H_{in}\left(f\right){|}^{2}} \end{array}$$

(6) $$\begin{array}{l} P_{ij}^{2}\left(f\right)=\frac{N_{ij}^{2}\left(f\right)}{N_{ii}\left(f\right)N_{jj}\left(f\right)} \end{array}$$

(7) $$S\left(f\right)=H\left(f\right)\;VH^{*}\;\left(f\right)$$

where $\delta_{ij}^{2}\left(f\right)$ is dDTF, $\eta_{ij}^{2}\left(f\right)$ is a different normalization of the DTF, and $P_{ij}^{2}\left(f\right)$ is partial coherence (pCoh). ***S***(***f***) is power spectra and V represents the variance of ***E***(***f***). ***N***_***ij***_(***f***) is obtained by removing the i-th row and j-th column of ***S***.

In this study, we used source information flow toolbox (SIFT) to compute dDTF, which is an electrophysiological information flow toolbox for EEGLAB. During the calculation, a rectangular window with a window length of 20 s and a step length of 5 s is applied. For the model order selection, four information criteria, including Akaike Information Criterion (AIC), Schwarz Bayes Criterion (SBC), Akaike's Final Prediction Error criterion (FPE), and Hannan-Quinn Criterion (HQ) were considered to determine a relatively suitable order. After completing the calculation of dDTF, two 22 by 22 adjacent connectivity matrixes (before and after intervention) were acquired for each subject.

### Graph Theory

A graph is a structure, which consists of a set of nodes and a set of lines called edges (Iakovidou, 2017). It can be used to mathematically represent a network (De Vico Fallani et al., 2007). When building a brain network based on graph theory, the nodes and edges need to be defined. In this study, we defined a single EEG channel as a node, and the weighted connections between different channels as an edge to construct a directed brain network.

Graph theory is an effective mathematical method for analyzing brain networks constructed based on EEG. In this study, five neurobiologically meaningful graph theory measures were used to investigate the topological organization of the brain network. Among them, the clustering coefficient and local efficiency are used to characterize the functional separation of brain networks, and the global efficiency characterizes the functional integration of brain networks. Node strength was used to measure the importance of a node in the brain network. Network density represents how sparse or dense brain networks are. All graph theory measures for each subject can be found in the Supplementary Material.

#### Threshold Selection

In the adjacent connectivity matrix, weighted connections exist between every possible pair of nodes, where there may be some spurious connections of low weight (Smith et al., 2015). Since spurious connections may cover up important connections between node pairs and affect subsequent graph theory analysis, it is necessary to threshold the weighted connection matrix. An objective threshold has not yet been proposed, so the determination of the threshold is arbitrary at present. Considering the rationality of the network, it is necessary to ensure that there are no isolated nodes in the network, and that the connection density should not be too high or too low when determining the threshold. Setting the threshold too high may lead to low connection density as well as isolated nodes, which may affect the integrity of the network. If the threshold is set too low, it may result in the ineffective removal of spurious connections.

We use the absolute value method to threshold the adjacency matrix. To build a reasonable network, we expand the range of thresholds for graph analysis, and the range of thresholds is 0.0075:0.0005:0.0095. Before subjecting the matrix values to a threshold, we set the weights on the diagonal of the adjacency matrix to 0 to ensure that all self-connections are removed. All weights below the threshold are then reset to 0. For weights higher than the threshold, it can be retained or set to 1. When we calculate the node strength, the connections higher than the threshold are retained. The calculation of the four graph parameters except node strength uses the binary connection matrix, that is, the connection above the threshold is set to 1.

#### Construction of Functional Brain Network

To more intuitively observe the changes in the number of effective connections between and within the hemisphere before and after the intervention, we averaged the weighted adjacency matrices of all subjects in each group. The thresholded average weighted connectivity matrix was used to construct the brain network connectivity map. We define each EEG channel as a node of the network. The size of the node indicates the node strength and the color of the node indicates the normalized node strength. Effective connections above the threshold are represented by three colors to distinguish connections in different regions, with red for connections within the ipsilesional hemisphere, blue for connections within the contralesional hemisphere, and purple for other connections. The thickness of the edges in the network represents the connection weights between the corresponding node pairs, and the direction of the arrow indicates the direction of information flow.

#### Node Strength

Node strength can be used to measure the importance or centrality of a node in the weighted network (Barrat et al., 2004). Its calculation takes into account the information of connection weights and the number of connections. Node strength is defined as the sum of inflow and outflow weights from a node (Iakovidou, 2017). The mathematical formulation for node strength is as follows (De et al., 2008):


(8) $$\begin{array}{l} S_{i}\;=\underset{j\epsilon V}{\sum }w_{ij}+\underset{j\epsilon V}{\sum }w_{ji} \end{array}$$

Where ***S***_***i***_ represents the node strength of node i. For a weighted connection matrix, ***w***_***ij***_ represents the connection weight from node j to node I, and ***w***_***ji***_ represents the connection weight from node i to node j. V = 1…N is the set of available nodes, and N is the number of nodes within the network.

#### Network Density

Network density is defined as the fraction of actual connections within the network to its possible maximal connections. The density ranges from 0 to 1, and a smaller value indicates a lower network density (De et al., 2008). The mathematical formulation for network density is as follows:


(9) $$\begin{array}{l} D\;=\;\frac{2L}{N\left(N-1\right)} \end{array}$$

where L is the actual connection in the network and N is the number of nodes.

#### Clustering Coefficient

The local clustering coefficient can be used to measure the neighborhood connectivity, which is defined as the ratio between the actual number of connections between all neighbor nodes and the maximal possible number of connections between these neighbor nodes (Kaiser, 2011). The magnitude of the local clustering coefficient is between 0 and 1. In an undirected network, the local clustering coefficient of node i is defined as:


(10) $$\begin{array}{l} C\left(i\right)=\frac{2t_{i}}{k_{i}\left(k_{i}-1\right)} \end{array}$$

where ***C***(***i***) is the local clustering coefficient of node i, ***k***_***i***_ is the degree of node i, ***t***_***i***_ is the number of triangles around node i. In a network, triangle is defined as a subgraph with 3 nodes and 3 connections (Jin et al., 2017).

The clustering coefficient of a network is the average of the local clustering coefficient of all nodes, which is generally considered to be a measure of the functional separation of brain networks. For an undirected network, the clustering coefficient is:


(11) $$\begin{array}{l} C\;=\;\frac{1}{N}\underset{i\in N}{\sum }C\left(i\right) \end{array}$$

where N is the number of nodes in a network. In a directed network, clustering coefficient is:


(12) $$\begin{array}{l} \vec{C}=\frac{1}{N}\underset{i\epsilon N}{\sum }\frac{{\vec{t}}_{i}}{\left(k_{i}^{out}+k_{i}^{in}\right)\left(k_{i}^{out}+k_{i}^{in}-1\right)-2\underset{j\in N}{\sum }a_{ij}a_{ji}} \end{array}$$

where ${\vec{t}}_{i}$ is the number of triangles around node i for a directed network, $k_{i}^{out}$ and $k_{i}^{in}$ represent the in-degree and out-degree of node i, respectively. For a binary connection matrix, ***a***_***ij***_ represents the connection state between node j and node i, and a value of 1 indicates that there is a connection, otherwise there is no connection.

#### Local Efficiency

Similar to the clustering coefficient, local efficiency can also be used to characterize the functional separation of brain networks. It is the mean of efficiencies of the local subgraphs of the first neighbors of each node i (Vragovi et al., 2005; Stam and Reijneveld, 2007). In an undirected network, the local efficiency of the entire network is:


(13) $$\begin{array}{l} E_{loc}=\frac{1}{N}\underset{i\epsilon N}{\sum }\frac{\sum_{j,h\in N,j\neq i,j\neq h,i\neq h}a_{ij}a_{ih}{\left[d_{jh}\left(N_{i}\right)\right]}^{-1}}{k_{i}\left(k_{i}-1\right)} \end{array}$$

where *d*_*jh*_(*N*_*i*_) is the shortest path length between nodes j and h (including only adjacencies of i.

For a directed network, the local efficiency is:


(14) $$\begin{array}{l} {\vec{E}}_{loc}=\frac{1}{2N}\underset{i\epsilon N}{\sum }\\ \frac{\sum_{j,h\in N,j\neq i,j\neq h,i\neq h}\left(a_{ij}+a_{ji}\right)\left(a_{ih}+a_{hi}\right)\left({\left[{\vec{d}}_{jh}\left(N_{i}\right)\right]}^{-1}+{\left[{\vec{d}}_{hj}\left(N_{i}\right)\right]}^{-1}\right)}{\left(k_{i}^{out}+k_{i}^{in}\right)\left(k_{i}^{out}+k_{i}^{in}-1\right)-2\underset{j\in N}{\sum }a_{ij}a_{ji}} \end{array}$$

#### Global Efficiency

The global efficiency is the average of inverse shortest path length (Rubinov and Sporns, 2010; Li et al., 2016; Hossein et al., 2018), which can be used to characterize the efficiency of informational exchange across the whole network (Ismail and Karwowski, 2020). The high global efficiency of a network means high information integration value (Hossein et al., 2018). For a directed network, the mathematical formulation for global efficiency is as follows:


(15) $$\begin{array}{l} {\vec{E}}_{glob}=\frac{1}{N}\underset{i\epsilon N}{\sum }\frac{\sum_{j\in N,j\neq i}{\left({\vec{d}}_{ij}\right)}^{-1}}{N-1} \end{array}$$

All the above graph theory measures were calculated based on the Brain Connectivity Toolbox (BCT) and scripts developed in Matlab R2020a.

### Statistical Analysis

To compare the differences between the experimental group and the control group before and after intervention, we conducted the statistical analysis of four graph theory measures. Shapiro–Wilk tests, which is an appropriate normality test in case of small sample sizes, were first used to check the normality of the variables. For variables conforming to the normal distribution, the paired sample *T*-tests were used for intra-group comparison before and after the intervention. The independent sample *T*-tests were applied to the difference between the experimental group and the control group. For variables that do not follow a normal distribution, we use non-parametric tests. The paired sample Wilcoxon signaled rank tests were used to compare the intra-group differences before and after the intervention. Mann Whitney U tests were performed between the two groups to determine whether the difference was statistically significant. All statistical tests took *p* < 0.05 as the criterion for significant differences.

## Results

### Clinical Scales Results

**Figure 4:**
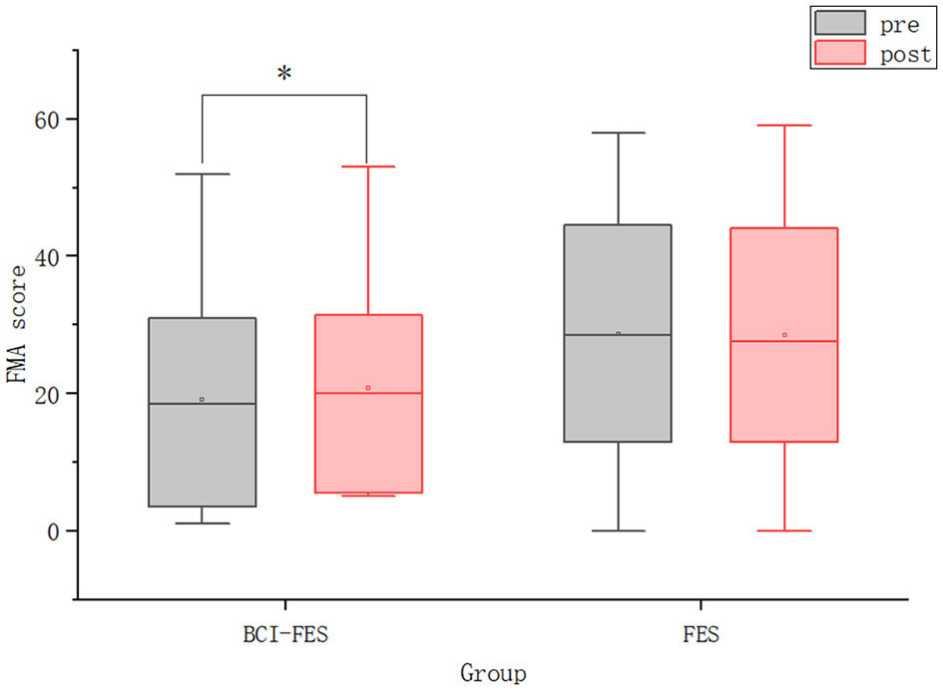
The FMA scores of patients in the two groups before and after the intervention. Hollow squares represent mean values. *p < 0.05.

We performed a statistical analysis of the FMA scores. As shown in Figure 4, there was no significant difference between the BCI–FES and FES groups before the intervention. This result indicates that there was no significant difference in the initial clinical status between the two groups, excluding the effect of the patients' initial condition. After the intervention, there was no intergroup difference in FMA scores. For within-group changes, there was a significant difference in FMA scores in the BCI–FES group before and after the intervention (pre: 19.17 ± 16.62, post: 20.75 ± 16.08, *p* = 0.008), indicating a significant improvement in motor function after the BCI rehabilitation training. However, no significant differences were found in the FES group before and after the intervention, which could indicate that the motor function of the patients was not significantly improved.

### Local Effective Connections

**Figure 5:**
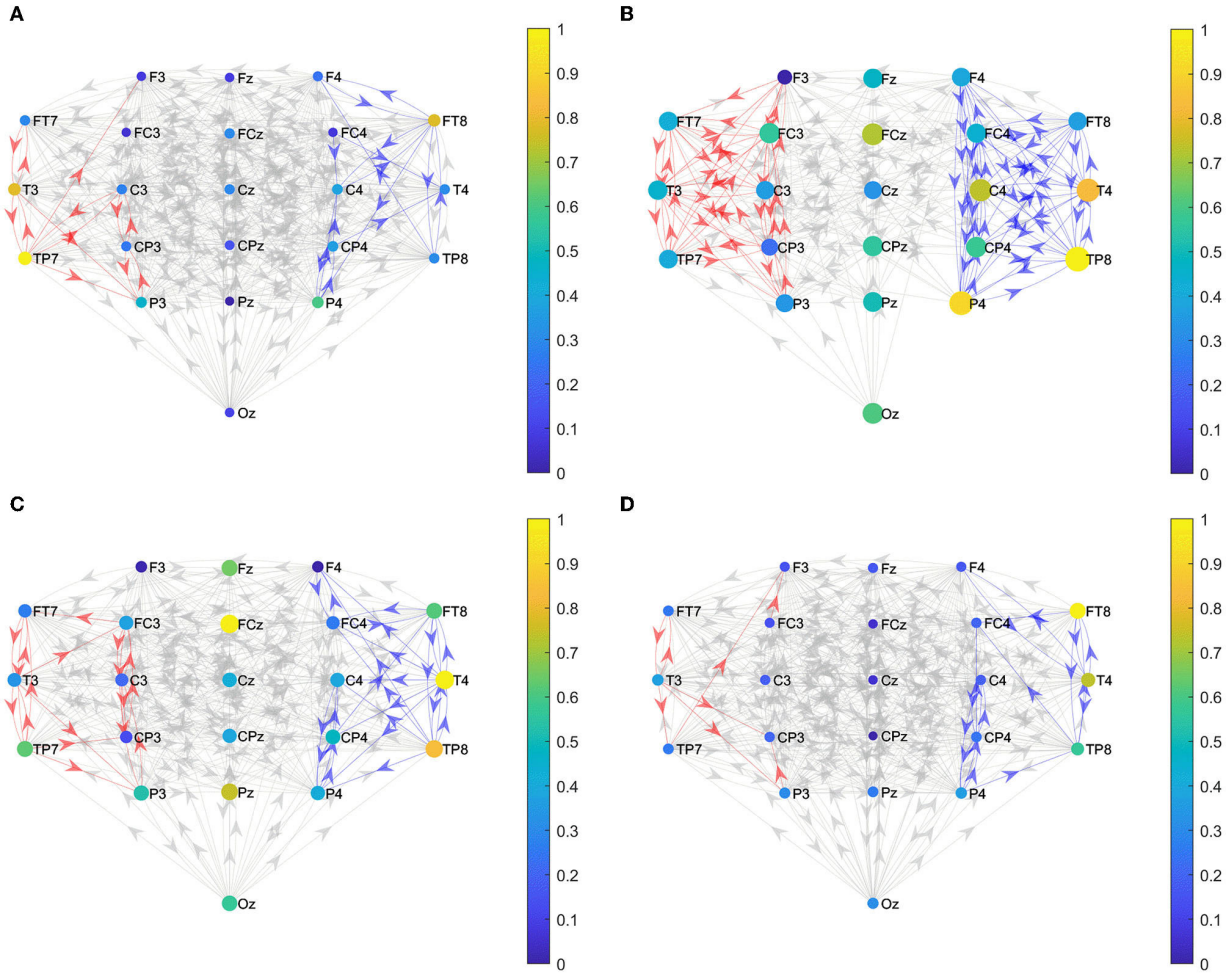
Effective connections within both hemispheres. **(A)** BCI–FES group before intervention; **(B)** BCI–FES group after intervention; **(C)** FES group before intervention; and **(D)** FES group after intervention. Gray edges indicate connections below the threshold, red edges represent effective connections within the ipsilesional hemisphere, blue edges indicate effective connections within the contralesional hemisphere, node size represents node strength, and node color indicates normalized node strength. The direction of the arrow indicates the direction of information flow.

**Figure 6:**
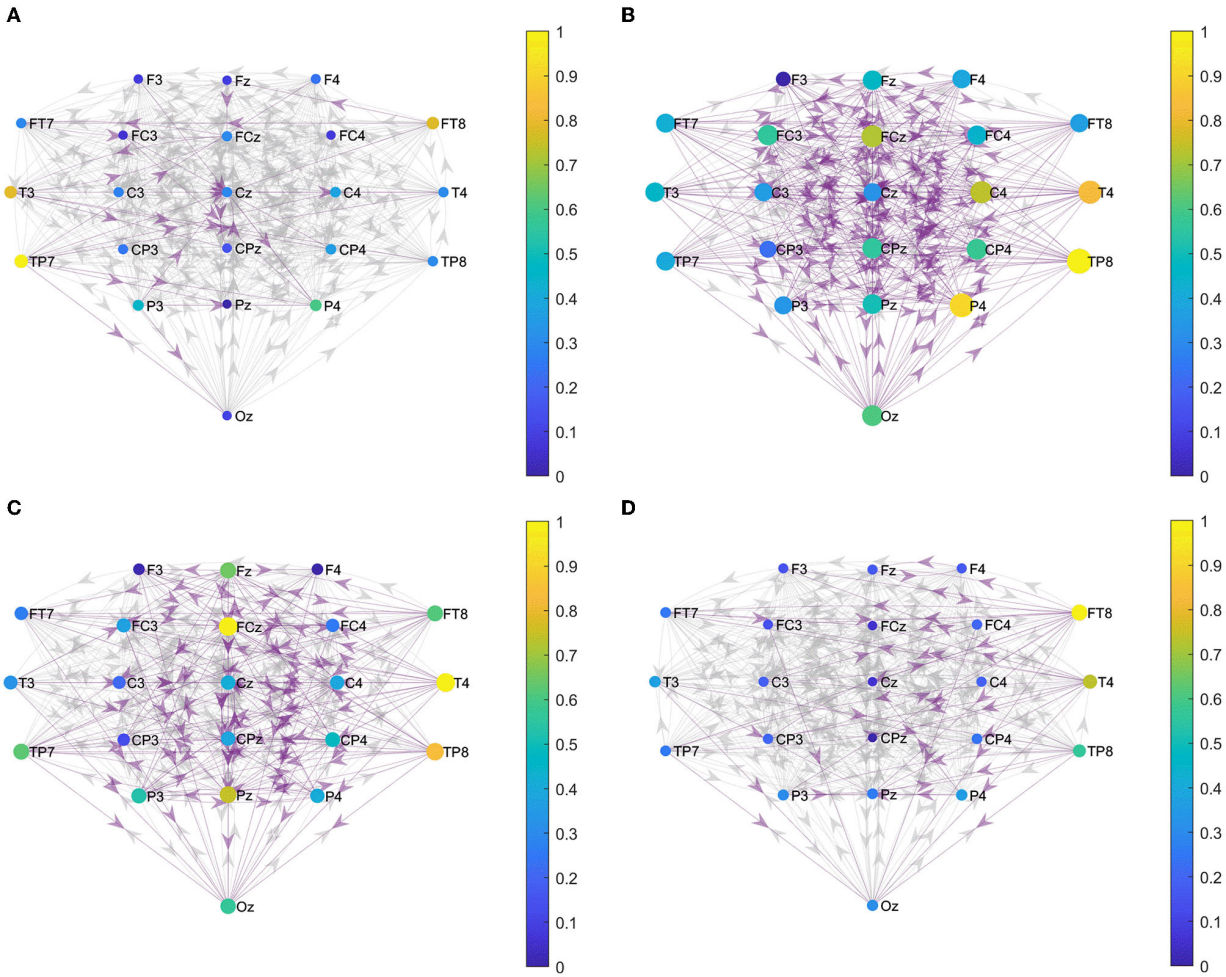
Effective connections between hemispheres. **(A)** BCI–FES group before intervention; **(B)** BCI–FES group after intervention; **(C)** FES group before intervention; and **(D)** FES group after intervention. Gray edges indicate connections below the threshold, red edges represent effective connections within the ipsilesional hemisphere, blue edges indicate effective connections within the contralesional hemisphere, node size represents node strength, and node color indicates normalized node strength. The direction of the arrow indicates the direction of information flow.

We constructed the functional brain networks before and after the intervention for the two groups within the alpha band with the threshold set to 0.0085. Figure 5 shows the effective connections within the two hemispheres of the brain network of the two groups before and after the intervention. Figure 6 shows the effective connections between the hemispheres in the brain network of the two groups of patients. The effective connections in the BCI–FES group after the intervention were much more than those before the intervention. Moreover, the effective connections within both hemispheres increased after the intervention, but the increase of effective connections within the contralesional hemisphere was more. Different from the BCI–FES group, the number of effective connections in the network decreased after the intervention in the FES group, and the number of effective connections within both the hemispheres decreased. For the average brain network in both groups, there were more effective connections within the contralesional hemisphere than within the ipsilesional hemisphere before and after the intervention. Before the intervention, the FES group had more effective connections than the BCI–FES group. But after rehabilitation training, the BCI–FES group had more effective connections in the brain network than the FES group.

### Nodal Metric Results

**Figure 7:**
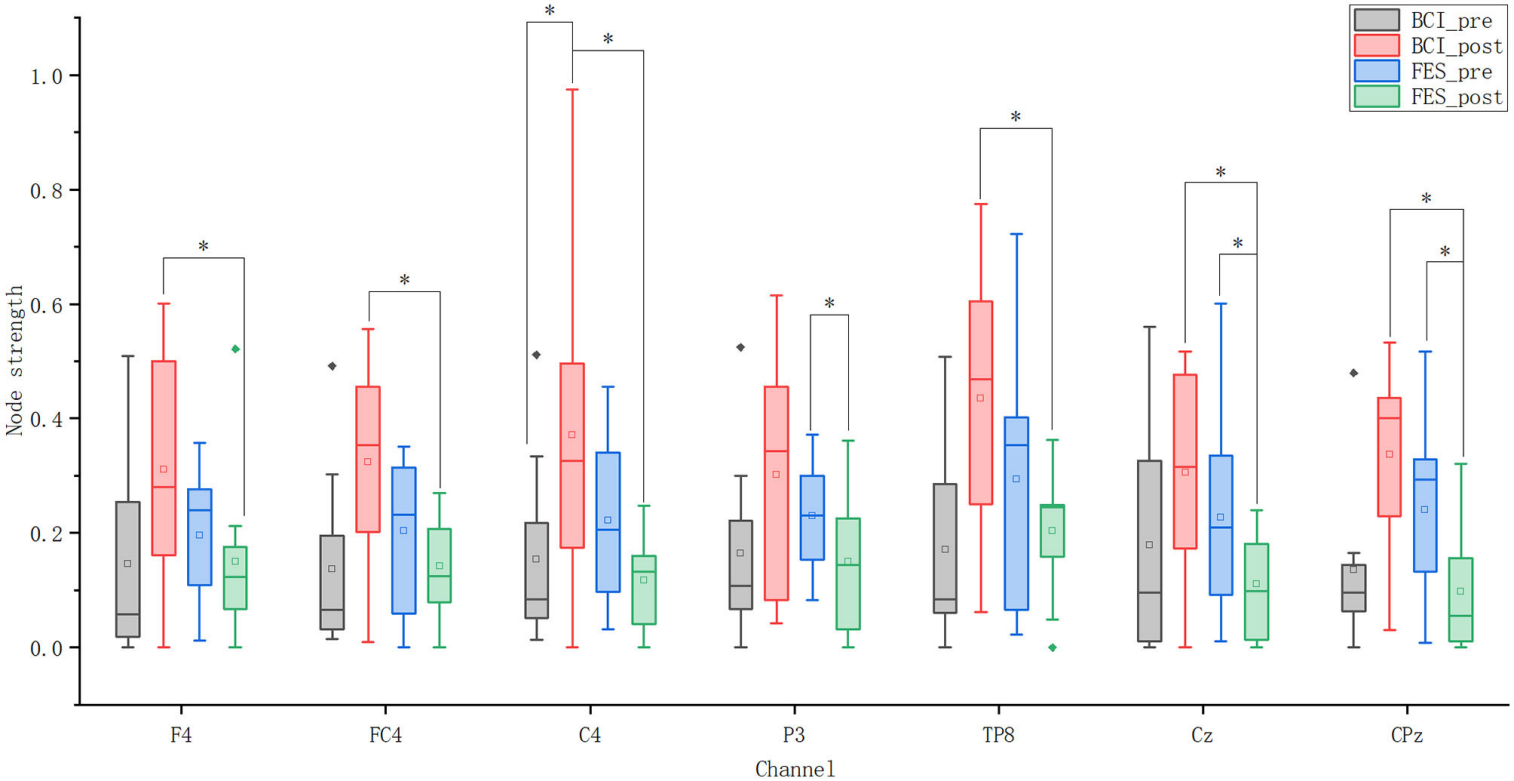
Node strength of seven nodes. Solid diamonds indicate outliers. Hollow squares represent mean values. *p < 0.05.

Figure 7 shows nodes with statistically significant differences in node strength between and within groups before and after the intervention. In terms of the node strength of these eight nodes, after the intervention, the node strength of the BCI–FES group increased, while the node strength of the FES group decreased. Before the intervention, the node strength of the FES group was higher than that of the experimental group, but the situation was reversed after the intervention.

For the BCI–FES group, statistical analysis showed that the node strength of C4 in the contralesional sensorimotor cortex was significantly increased (pre-C4: 0.154 ± 0.175, post-C4: 0.424 ± 0.285, *p* = 0.035). For the FES group, node strength in the ipsilesional hemisphere (P3) (pre-P3: 0.23 ± 0.103, post-P3: 0.169 ± 0.131, *p* = 0.049) and central region (Cz, CPz) (pre-Cz: 0.228 ± 0.183, post-Cz: 0.125 ± 0.088, *p* = 0.029) (pre-CPz: 0.24 ± 0.157, post-CPz: 0.126 ± 0.109, *p* = 0.043) decreased significantly after rehabilitation training. Differences in node strength between the two groups after the intervention were compared. It can be found that the node strength in the contralateral hemisphere (F4, FC4, C4, TP8) (BCI–FES-F4: 0.355 ± 0.191, FES-F4: 0.169 ± 0.156, *p* = 0.032) (BCI–FES-FC4: 0.32 ± 0.188, FES-FC4: 0.16 ± 0.088, *p* = 0.042) (BCI–FES-C4: 0.424 ± 0.285, FES-C4: 0.132 ± 0.077, *p* = 0.035) (BCI–FES-TP8: 0.435 ± 0.252, FES-TP8: 0.229 ± 0.101, *p* = 0.049) and the central region (Cz, CPz) (BCI–FES-Cz: 0.349 ± 0.152, FES-Cz: 0.125 ± 0.088, *p* = 0.003) (BCI–FES-CPz: 0.337 ± 0.173, FES-CPz: 0.126 ± 0.109, *p* = 0.016) of the BCI–FES group was significantly higher than that of the FES group.

### Global Metric Results

#### Network Density

**Figure 8:**
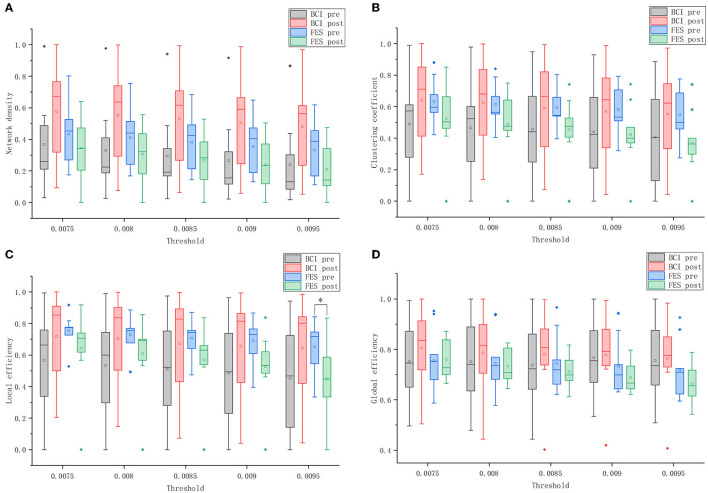
Global graph theory measures of the brain networks of the two groups before and after the intervention at different thresholds. **(A)** network density; **(B)** clustering coefficient; **(C)** local efficiency; and **(D)** global efficiency. Solid diamonds indicate outliers. Hollow squares represent mean values. *p < 0.05.

Figure 8A shows that the network density of both groups decreased as the threshold increased. When the threshold value was constant, the network density of the BCI–FES group increased after the intervention, while the network density of the FES group decreased. Comparing the changes between groups, it can be found that the network density of the FES group was higher than that of the BCI–FES group before the intervention. However, after the intervention, the network density of the BCI–FES group was higher than that of the FES group after the intervention. The results of the statistical analysis show that there were no statistically significant differences between and within groups before and after the intervention.

#### Clustering Coefficient

Figure 8B shows the clustering coefficients of the BCI–FES and FES groups before and after the intervention at different thresholds. Comparing the changes in the mean values of clustering coefficients before and after the intervention, it can be found that the BCI–FES group showed an increasing trend, while the FES group showed a decreasing trend. The differences in mean clustering coefficients between the two groups were compared. It can be found that before the intervention, the FES group had a higher clustering coefficient, but after the intervention, the clustering coefficient of the BCI–FES group was higher than that of the FES group. The comparisons between and within groups at different thresholds did not show statistically significant differences.

#### Local Efficiency

As is shown in Figure 8C, the mean value of the local efficiency of all subjects after the intervention tended to increase in the BCI–FES group, with no statistically significant differences. In the threshold interval, the local efficiency of the FES group showed a decreasing trend after the intervention, and when the threshold value was set to 0.0095, the mean value of local efficiency in the FES group after the intervention was significantly lower than that before the intervention (pre: 0.651 ± 0.167, post: 0.448 ± 0.238, *p* = 0.049).

Comparing the changes between groups, it can be found that the mean local efficiency of the FES group was higher than that of the BCI–FES group before the intervention. However, the mean local efficiency of the BCI–FES group outperformed the control group after the intervention. There were no statistically significant differences between the groups before and after the intervention.

#### Global Efficiency

Figure 8D shows that within the threshold interval, the mean of global efficiency of all subjects in the BCI–FES group improved after the intervention. For the FES group, a decreasing trend in the mean value of global efficiency of all subjects after the intervention could be observed. There was no statistically significant differences within the group before and after the intervention for both of the two groups.

Moreover, the mean values of global efficiency were similar between the two groups before the intervention. But the global efficiency of the BCI–FES group was higher than that of the FES group after the intervention. There were no statistically significant differences between the two groups before and after the intervention.

## Discussion

### Global Alterations of Brain Network

We used four-graph theory measures, including network density, clustering coefficient, local efficiency, and global efficiency, to compare and analyze the overall changes in the brain network of patients with stroke before and after the intervention. After the intervention, the four measures were higher in the BCI–FES group than before the intervention, and the opposite changes were observed in the FES group. In terms of the mean values of the four measures, the FES group was higher than or similar to the BCI–FES group before the intervention, but the BCI–FES group was higher than the FES group after the intervention. These experimental results suggest that patients in the BCI–FES group showed positive changes in their brain networks after the intervention, and these changes were consistent with the changes in the FMA scores. The FES group did not show statistically significant differences in FMA scores before and after the intervention, but the overall network parameters in the control group tended to decrease after the intervention (no statistically significant differences).

This change in the FES group was not surprising due to our experimental design. We recruited subjects who were elderly patients with chronic stroke with a disease duration of 1 or 2 years. Usually, 3 or 6 months after stroke, the recoveries for the patients reach a plateau (Aziz, 2010), as evidenced by patients becoming more chronic and/or not responding positively to motor rehabilitation (Page et al., 2004). Six to twelve months after stroke, the potential for recovery substantially diminishes according to the conventional clinical wisdom (Soekadar et al., 2015). Moreover, the chronic phase of stroke is usually considered to be the terminal stage when the adaptive regenerative process stops (Barios et al., 2021). Page et al. (2004) pointed out that a contributing factor to the plateau in post-stroke rehabilitation is neuromuscular adaptation to a standardized outpatient exercise regimen. Based on this, it is understandable that there was no statistically significant difference in FMA scores before and after the intervention in the control group. Our experimental results show that FES combined with conventional rehabilitation training has no significant effect on the rehabilitation of patients with chronic stroke.

Different from the FES group, the clinical scale results show that the motor function of patients in the BCI–FES group was significantly improved. Moreover, the brain network showed positive changes. The clustering coefficient, local efficiency, and global efficiency of the BCI–FES group after intervention were higher than those before intervention. This result indicates that brain networks of patients with chronic stroke in the BCI–FES group showed a higher capacity for separation and integration after the intervention, with relatively easy information exchange between brain regions. All these experimental results demonstrate that BCI–FES intervention therapy plays an effective and positive role in the recovery of patients with chronic stroke, which is consistent with the findings of some previous studies (Broetz et al., 2010; Ramos-Murguialday et al., 2013; Mukaino et al., 2014). Although recovery is clinically considered to substantially diminish in patients with chronic stroke, several recent studies have shown that non-invasive treatment strategies used for rehabilitation, including constraint-induced movement therapy (CIMT) (Sirtori et al., 2009), functional stimulation (Biasiucci et al., 2018), and BCI (Ramos-Murguialday et al., 2013) are effective in the rehabilitation of chronic stroke. The success of these strategies suggests that recovery from chronic stroke depends to some extent on learning and environmental conditions, and the results of these studies provide evidence for the concept of Page et al. (2004) that new treatment options can facilitate recovery as well as overcome neuromuscular adaptations.

### Regional Alterations of Brain Network

We compared the changes in node strength and the number of effective connections within the hemisphere before and after the intervention in two groups of patients with chronic stroke. The results show that the node strengths in the contralesional hemisphere and the central region were significantly higher in the BCI–FES group than in the FES group after the intervention. In addition, the number of effective connections in the contralesional hemisphere increased in the BCI–FES group and decreased in the FES group after the intervention. The changes in node strength and number of effective connections indicated that the functional connectivity in the contralesional hemisphere was enhanced in the BCI–FES group compared with the FES group.

After intervention, the node strength and the number of effective connections in the FES group were both lower than before the intervention. One possible explanation for this result is that our subjects were all elderly patients with chronic stroke, and aging negatively affects brain networks. Since motor function in the FES group did not improve as significantly as in the BCI–FES group, the brain network in the FES group did not show positive changes with the improvement of motor function. A graph theory-based study of brain networks has shown that normal aging results in a certain degree of damage to brain functional networks (Achard and Bullmore, 2007). Based on the above conclusions and the fact that our subjects were all elderly, we speculated that the decrease in brain connections in the FES group might be caused by normal aging.

One possible explanation for the findings of enhanced functional connectivity in the contralateral hemisphere in BCI–FES group of patients with chronic stroke is that the contralesional hemisphere compensates for the activity of the ipsilesional hemisphere. The contralateral hemisphere of some patients with chronic stroke retains compensatory mechanisms that are absent in others (Barios et al., 2021). It was found that patients with chronic stroke had increased activation in the contralesional hemisphere during movement of the affected hand (Christian et al., 2006; Barios et al., 2021). After receiving BCI–FES intervention, the number of effective connections in the contralesional hemisphere was higher than that in the ipsilesional hemisphere in patients with chronic stroke. Furthermore, the node strength of the C4 node in the contralesional sensorimotor cortical area was significantly enhanced. This result suggests that nodes in the contralesional hemispheric sensorimotor area of patients with chronic stroke are more important in the transmission of information throughout the brain network after receiving BCI–FES rehabilitation training compared to before the intervention. After the intervention, the activation of the contralesional hemisphere was increased in the BCI–FES group. But patients with chronic stroke treated with the FES intervention did not show this increased activation of the contralesional hemisphere. Therefore, we believe that the increased activation of the contralesional hemisphere in the BCI–FES group may be caused by the BCI–FES rehabilitation training. Similarly, Sun et al. (2017) found that BCI rehabilitation resulted in a significant increase in EEG fApEn in the central region of the contralesional hemisphere in patients with chronic stroke. Previous studies have shown that changes in the activity of the contralesional hemisphere in patients with chronic stroke are associated with functional recovery (Barios et al., 2021). Patients with chronic stroke who received BCI–FES intervention had significantly improved motor function. However, the motor function of patients with chronic stroke in the FES group did not improve significantly. Based on this, we speculate that the improved motor function in the BCI–FES group may be related to the increased activation of the contralesional hemisphere. The results of a previous study also suggest that BCI rehabilitation can exploit the plasticity of the contralesional corticospinal tracts to change them from a deleterious to a compensatory effect (Young et al., 2016). Activation of the contralateral hemisphere is increased, and compensatory action leads to improved motor function. In conclusion, we suggest that BCI–FES rehabilitation training played a role in inducing increased activation of the contralesional hemisphere and facilitating the activity of the contralesional hemisphere to compensate for the affected hemisphere.

### Conclusion

For chronic stroke prognosis, BCI combined with external devices may be more effective than traditional rehabilitation strategies, but it is lacks a comprehensive assessment of neurological changes associated with functional rehabilitation. In this study, we used EEG-based brain network analysis to comprehensively and quantitatively investigate the changes in brain activity of patients with chronic stroke induced by BCI–FES rehabilitation training. The clinical scale results show that the patients' FMA scores improved significantly after BCI–FES rehabilitation training. Also, according to the graph theory analysis, both the functional integration and functional separation of the brain network of the patients in the BCI–FES group were improved. These findings demonstrate that BCI–FES rehabilitation training can effectively improve motor function in patients with chronic stroke, which is consistent with previous studies. In addition, we also found that the node strength in the contralesional hemisphere was significantly higher in BCI–FES patients than in the FES group. The increased importance of the contralesional hemisphere in network information transmission suggests that BCI–FES rehabilitation training may promote compensatory activity in the contralesional hemisphere. In conclusion, the findings of our study demonstrate the effectiveness of BCI–FES rehabilitation training in the prognosis of patients with chronic stroke. It is also pointed out that BCI–FES rehabilitation training improves the efficiency of brain network information transmission and promotes the compensatory effect of the contralesional hemisphere.

Our study provides a new perspective for a comprehensive assessment of changes in brain activity induced by BCI–FES rehabilitation training. However, due to the limited number of EEG samples, the statistical analysis of some graph theory measures did not show significant differences. Future studies need to evaluate more patients with chronic stroke to validate the conclusions of this study.

## Data Availability Statement

The original contributions presented in the study are included in the article/Supplementary Material, further inquiries can be directed to the corresponding authors.

## Ethics Statement

The studies involving human participants were reviewed and approved by Ethics Committee of Huashan Hospital. The patients/participants provided their written informed consent to participate in this study.

## Author Contributions

JJ, YX, and SC contributed to the design of the experiment. SC and YJ contributed to the data acquisition. GZ, ZS, and XK analyzed data. GZ, SC, and XK wrote the first draft of the manuscript. GZ, SC, JW, LN, JB, JJ, and XK contributed to manuscript revision. All authors contributed to the article and approved the submitted version.

## Funding

This work was supported in part by the National Natural Science Foundation of China, Grant Nos. 61904038 and U1913216; National Key R&D Program of China, Grant Nos. 2021YFC0122702 and 2018YFC2002300; Shanghai Sailing Program, Grant Nos. 19YF1403600 and 22YF1404200; Shanghai Municipal Science and Technology Commission, Grant Nos. 19441907600, 19441908200, and 19511132000; Opening Project of Zhejiang Lab, Grant No. 2021MC0AB01; Fudan University-CIOMP Joint Fund, Grant No. FC2019-002; Opening Project of Shanghai Robot R&D and Transformation Functional Platform, Grant No. KEH2310024; Ji Hua Laboratory, Grant Nos. X190021TB190 and X190021TB193; National Natural Integration Project, Grant No. 91948302; Shanghai Municipal Science and Technology Major Project, Grant Nos. 2021SHZDZX0103 and 2018SHZDZX01.

## Conflict of Interest

The authors declare that the research was conducted in the absence of any commercial or financial relationships that could be construed as a potential conflict of interest.

## Publisher's Note

All claims expressed in this article are solely those of the authors and do not necessarily represent those of their affiliated organizations, or those of the publisher, the editors and the reviewers. Any product that may be evaluated in this article, or claim that may be made by its manufacturer, is not guaranteed or endorsed by the publisher.
